# Isolated methylmalonic acidemia in Mexico: Genotypic spectrum, report of two novel MMUT variants and a possible synergistic heterozygosity effect

**DOI:** 10.1016/j.ymgmr.2024.101155

**Published:** 2024-10-16

**Authors:** Cynthia Fernández-Lainez, Marcela Vela-Amieva, Miriam Reyna-Fabián, Liliana Fernández-Hernández, Sara Guillén-López, Lizbeth López-Mejía, Miguel Ángel Alcántara-Ortigoza, Ariadna González-del Angel, Rosa Itzel Carrillo-Nieto, Enrique Ortega-Valdez, Mauricio Rojas-Maruri, Cecilia Ridaura-Sanz

**Affiliations:** a Laboratorio de Errores Innatos del Metabolismo y Tamiz, Instituto Nacional de Pediatría, Mexico; b Laboratorio de Biología Molecular, Instituto Nacional de Pediatría, Mexico; c Facultad de Ciencias, UNAM, Mexico; d Departamento de Patología, Instituto Nacional de Pediatría, Mexico

**Keywords:** Double heterozygous, synergistic heterozygosity, methylmalonic acid, Inborn errors of metabolism, Propionate defects, Newborn screening

## Abstract

Isolated methylmalonic acidemia (iMMA) is a group of monogenic metabolic disorders affecting methylmalonate and cobalamin metabolism. Five iMMA-responsible genes have been described to date: *MMUT* (MIM *609058), *MMAA* (MIM *607481, *MMAB* (MIM *607568), *MMADHC* (MIM *611935), and *MCEE* (MIM *608419). Although iMMA is the most common form of organic acidemia reported in Mexico, its genotypic spectrum is still largely unknown. We performed a clinical exome analysis on 42 unrelated Mexican patients with iMMA. *MMUT* deficiency accounted for 73.8 % of all cases, followed by *MMAA* (14.2 %), *MMAB* (7.2 %), and *MMADHC* (2.4 %) deficiencies. One patient presented *MMUT* and *MMAA* double heterozygosity, which should be further experimentally confirmed to prove that synergistic heterozygosity could be another inheritance mechanism in iMMA. The most frequent *MMUT* genotype involved the Hispanic variant NM_000255.4:c. [322C > T];[322C > T] or p.[Arg108Cys];[Arg108Cys] (14.3 %). Two novel *MMUT* variants, NM_000255.4:c.589G > A or p.(Ala197Thr) and c.1476C > A or p.(Tyr492*), were identified in a deceased newborn presenting the neonatal-onset severe form of the disease. *In silico* protein modeling of the p.(Arg108Cys) and novel p.(Ala197Thr) *MMUT* variants suggested disruption of the substrate-binding and catalytic domains of the protein, respectively. This study expands the current knowledge on the molecular spectrum of iMMA in the Mexican population and reinforces the importance of genetic analysis in guiding clinical management.

## 1 Introduction

Isolated methylmalonic acidemia (iMMA) refers to a complex and heterogeneous group of monogenic disorders affecting the metabolism of methylmalonate and cobalamin (Cbl, vitamin B12), characterized by elevated concentrations of methylmalonic acid (MMA) in blood and urine [1,2], which arises from a partial or complete deficiency of the enzyme methylmalonyl-CoA mutase (MUT), a defect in the transport or synthesis of its cofactor 5-deoxyadenosyl-cobalamin (AdoCbl) (cblA, cblB, cblD-MMA), or a defect in the enzyme methylmalonyl-CoA epimerase (MCEE) [3,4].

iMMA is one of the most common organic acidemias worldwide, and its heterogeneous clinical presentation can manifest at any age, from newborns to adults [5]. Clinically, iMMA is a multisystemic disease that affects several organs, including the brain, heart, gastrointestinal tract, pancreas, kidney, eye, and the hematological, musculoskeletal, and immune systems [1,4,6], and the disease confers a high risk of developing liver neoplasms [7]. iMMA is associated with high morbidity and mortality, and current treatments remain insufficient, highlighting its status as a difficult-to-treat condition and a top priority for continued research [5].

The physiopathology of iMMA is complex and incompletely understood [4]; it involves a significant biochemical imbalance, including metabolic acidosis, hyperammonemia, hypoglycemia, hyperketonemia, and hyperglycinemia; thus, it is considered a metabolic catastrophe mainly due to the disruption of cellular energy, including alterations in the Krebs and urea cycles [4,8]. Additionally, lysosomal–autophagy dysfunctions have been detected in MUT-deficient cells [9].

Blood methylmalonic acid and propionyl carnitine (C3) are considered the canonical biomarkers of iMMA [10]. The accumulation of C3 in the blood facilitates disease detection by tandem mass spectrometry (MS/MS), which is now included in most newborn screening panels worldwide [11]. Other diagnostic and prognostic markers, such as fibroblast growth factor-21, growth differentiation factor-15, and lipocalin-2, have also been described [10].

Wide genetic heterogeneity underlies iMMA, with five responsible genes described to date: *MMUT* (MIM *609058), *MMAA* (MIM *607481), *MMAB* (MIM *607568), *MMADHC* (MIM *611935), and *MCEE* (MIM *608419) [1]. According to the LOVD database (https://www.lovd.nl/, accessed 03/07/2023), a total of 344 unique variants are associated with iMMA, with *MMUT* variants being the most common (233/344, 67.73 %), followed by *MMAB* (68/344, 19.76 %), *MMAA* (18/344, 5.23 %), *MMADHC* (16/344, 4.65 %) and *MCEE* (9/344, 2.61 %).

Although iMMA is the most common form of organic acidemia diagnosed in Mexico, its genotypic spectrum has not yet been fully characterized [12]. In 2006, Worgan et al. studied patients with iMMA from several ethnic groups, including Hispanics living in Canada. Their findings revealed that nearly all Mexican individuals with MUT deficiency (MUTd) carried the novel *MMUT* variant c.322C > T or p.(Arg108Cys) [rs121918257], as well as the known variants c.280G > A or p.(Gly94Arg) [rs727504022], c.1022dupA or p.(Asn341Lysfs*20) [rs752898811], and c.970G > A or p.(Ala324Thr) [rs780387525]. These variants were subsequently referred to as “Hispanic” variants [13]. However, the genotypic spectra of the other four genes responsible for iMMA have not been investigated in Mexican patients. Therefore, this study aims to a) describe the complete genotypic spectrum of Mexican patients with iMMA; b) determine the proportion of individuals affected by each genotype of iMMA (*MMUT, MCEE MMAA, MMAB, or MMADHC*); c) analyze clinical characteristics to establish a possible phenotype–genotype correlation of the Hispanic *MMUT* variants described by Worgan in our cohort; d) perform *in silico* structural protein modeling of the novel *MMUT* missense variant p.(Ala197Thr) and the most frequent *MMUT* variant p.(Arg108Cys); and e) present the clinical and histopathological features of a patient with a novel *MMUT* genotype.

## 2 Methods

### 2.1 Subjects

From a cohort of pediatric patients with inborn errors of intermediary metabolism at the National Institute of Pediatrics, we included 42 patients (21 males and 21 females, mean age at diagnosis: 9.5 months, range: 7 days to 53 months) with a biochemical diagnosis of iMMA. Clinical data were recorded at the time of diagnosis for each patient, including age at symptom onset, age at diagnosis, demographic information, phenotype description, treatment, outcomes, and the diagnostic odyssey, defined as the time between symptom onset and biochemical diagnosis.

### 2.2 Biochemical diagnosis

Biochemical diagnosis was performed by analyzing the profiles of amino acids and acylcarnitines from dried blood spots by tandem mass spectrometry (MS/MS). Additionally, urinary methylmalonic acid levels were determined through gas chromatography coupled with mass spectrometry (GC/MS), following the methodology previously reported by Ibarra et al. 2021 [14]. A case of iMMA was defined by elevated blood concentrations of propionyl carnitine (C3), increased urinary concentrations of methylmalonic and methyl citric acids and propionyl glycine, with normal plasma homocysteine levels. Glycine blood concentrations were also considered, although normal levels do not exclude an iMMA case. Blood concentrations of homocysteine were measured by high-performance liquid chromatography (HPLC) to exclude cobalamin defects according to the Manoli protocol [10]. Given that the focus of the study was iMMA, we excluded patients with Cbl defects presenting elevated methylmalonic acid levels and high blood concentrations of homocysteine.

### 2.3 Clinical exome analysis and variant interpretation

Genomic DNA was extracted from peripheral blood leukocytes or buccal swab cells using a commercially available silica-based kit (QIAamp DNA Blood Mini Kit, Hilden, Germany) according to the manufacturer's protocol. Genetic testing was performed in a clinical exome sequencing study. The genomic libraries were prepared using the Flex for Enrichment kit, and the exonic regions were captured using the TruSight One Expanded kit. The libraries were sequenced through massive parallel sequencing (paired-end 2 × 150 bp) using Illumina technology. The sequenced data were evaluated for quality control using the FastQC program (version 0.12.0, https://www.bioinformatics.babraham.ac.uk/projects). The reads were aligned with BWA [15] against the human genome version GRCh38 (http://www.ncbi.nlm.nih.gov/grc/human). Variant calling was performed with GATK4 [16], the effects of the variants were assessed using SnpEff [17], and the selected variants were inspected in IGV [18]. Copy number variants (CNVs) were analyzed with the Franklin Genoox tool (https://franklin.genoox.com/clinical-db/home), and mitochondrial DNA was also analyzed.

The pathogenicity of each variant was evaluated according to the American College of Medical Genetics and Genomics and the Association for Molecular Pathology (ACMG/AMP) guidelines [19].

### 2.4 *In silico* protein modeling for structural analysis of *MMUT* variants

Structural analysis of two missense *MMUT* variants, p.(Ala197Thr) and p.(Arg108Cys), was performed based on the MUT crystallographic structure previously reported by Froese et al., 2010 (PDB Code: 2XIJ [20]). *In silico* mutagenesis analysis was performed with PyMOL software (PyMOL Molecular Graphics System, version 2.0 Schrödinger, LLC [21]) to identify the localization of the involved amino acid residues and to hypothesize the potentially deleterious effects of these amino acid changes on the protein structure.

### 2.5 Statistical analyses

Clinical data were retrospectively collected by reviewing medical records. The data are presented in tables and heatmap figures constructed using Microsoft Excel and GraphPad Prism software (version 9.4.1, San Diego, CA, USA). Descriptive statistics were performed, and the data distribution was assessed using the Shapiro–Wilk test. For parametric data, one-way ANOVA, mixed-effects analysis with Geisser–Greenhouse correction, and Dunnett's multiple comparison tests were conducted, and the results are presented as the means ± SDs. For nonparametric data, the Mann–Whitney *U* test was used, followed by Dunn's multiple comparisons adjustment test, with results presented as medians (Q1–Q3). Fisher's exact test was used to investigate significant differences in the proportions of qualitative variables. GraphPad Prism software (version 9.4.1, San Diego, CA, USA) was used for all the statistical tests.

### 2.6 Ethical considerations

This study was approved by the research, ethics, and biosafety institutional committees (protocol reference number 2024/032). Written informed consent was obtained from the patient's parents or legal guardians, secondary findings were not sought. All patients received pre- and post-clinical exome genetic counseling and medical follow-up.

## 3 Results

### 3.1 Genotypic spectrum of iMMA in Mexico

**Fig. 1:**
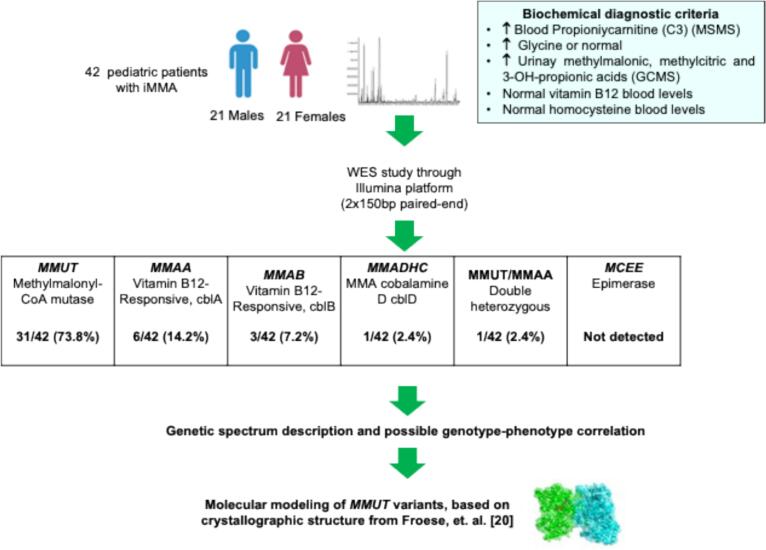
General scheme of the study.

After clinical exome analyses, the average coverage depth exceeded 99.8 % of the targeted regions, with a minimum of 20 reads for all patients. The most common form of iMMA was MUT deficiency (MUTd), accounting for 31 out of 42 patients (Patient IDs iMMA01–iMMA08, iMMA10–iMMA32) (73.8 %), followed by MMAA deficiency (MMAAd) in 6 out of 42 patients (Patient IDs iMMA33–iMMA38) (14.2 %), MMAB deficiency (MMABd) in 3 out of 42 patients (Patient IDs iMMA39–iMMA41) (7.2 %), MMADHC deficiency (MMADHCd) in 1 out of 42 patients (Patient ID iMMA42) (2.4 %), and 1 out of the 42 patients (Patient ID iMMA09) (2.4 %) who presented double heterozygosity with one variant in *MMUT* and one in *MMAA* (Table 1, Fig. 1).

**Fig. 2:**
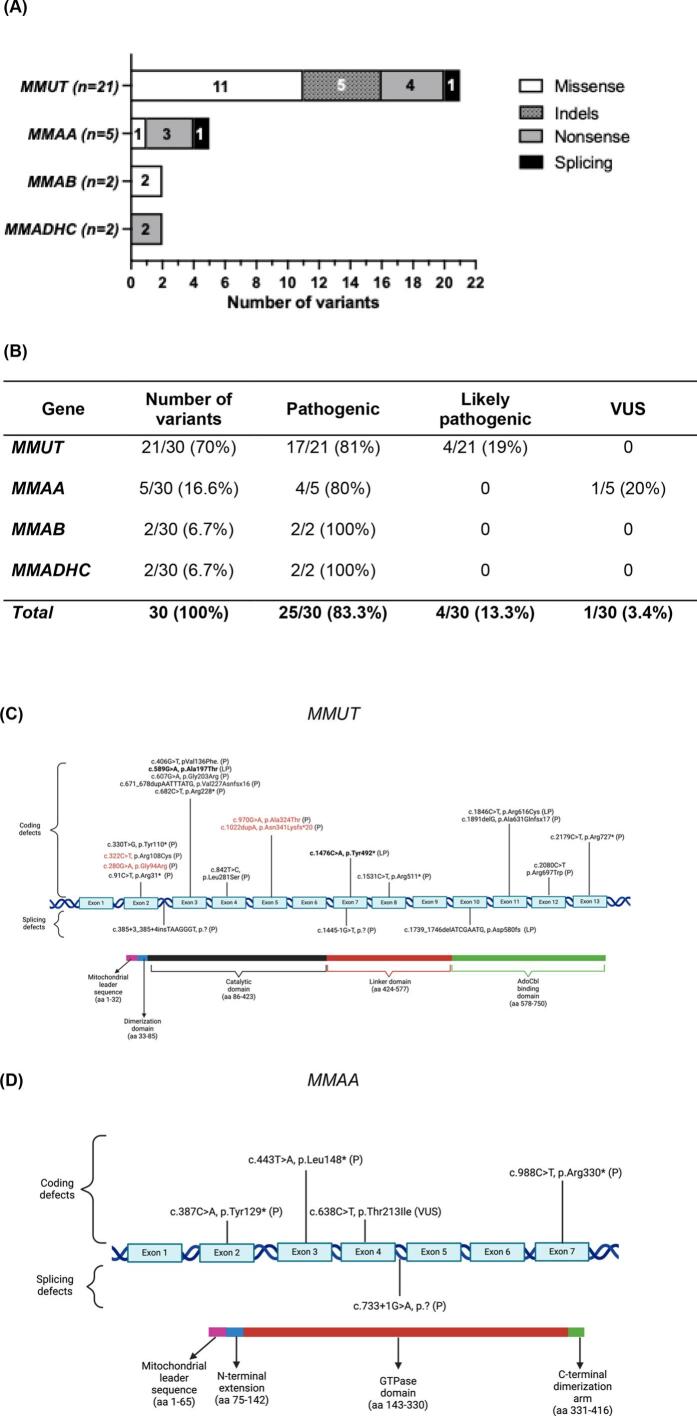
Mutational spectrum in Mexican patients with iMMA. (A) Distribution and gene variant classification accordingly to their predicted effect on protein. B) Distribution and classification of variants according to their pathogenicity. C) Variants located in *MMUT*. Protein domains are described in the lower part of the figure. Variants previously found in the Hispanic population, high frequency, or in the homozygous state, are highlighted in red, blue, or in bold, respectively. The amino acid residues by domain of MUT are based on Acquaviva, et al. [3]. D) Variants located in the *MMAA* gene. Protein domains are described in the lower part of the figure. The amino acid residues by domain of MMAA are based on Froese, et al. [20]. Classification of variants in *MMAB* and *MMADHC* are not illustrated since only two variants were found. P = pathogenic variant; LP = likely pathogenic variant; VUS = Uncertain significance variant.

A total of 30 different variants were identified across the various types of iMMA. Missense variants were most common in *MMAB* (2 out of 2, 100 %) and *MMUT* (11 out of 21, 52.4 %), whereas nonsense variants were more prevalent in *MMADHC* (2 out of 2, 100 %) and *MMAA* (3 out of 5, 60 %) (Fig. 2A). The least frequent variants were indels, which were identified exclusively in the *MMUT* gene (5 out of 21, 23.8 %), followed by splicing variants, which were present in *MMUT* (1 out of 25, 4.76 %) and *MMAA* (1 out of 5, 20 %).

According to the assessment of pathogenicity, most of the identified variants were pathogenic (25 out of 30, 83.3 %), followed by likely pathogenic variants (4 out of 30, 13.3 %); only one variant of uncertain significance (VUS) was documented (3.4 %) (Fig. 2B).

Two novel *MMUT* variants that have not yet been reported in either the literature or public databases were found in a compound heterozygous state (ID iMMA26). The first is a missense variant located in exon 3: c.589G > A or p.(Ala197Thr), and the second is a nonsense variant in exon 7: c.1476C > A or p.(Tyr492*) (Table 1). Nine out of the 21 variants affected the catalytic domain of the MUT protein (42 %), followed by 3 out of 21 variants (14.3 %) located in the dimerization domain of the MUT protein (Fig. 2C). The five variants found in *MMAA* are shown in Fig. 2D, three of which are in the GTPase domain of the protein.

Thirty different genotypes were found among the studied patients. Most of them were found in *MMUT* (21 out of 30, 70 %), followed by *MMAA* (5 out of 30, 16.6 %). The most frequent genotype found was c.[322C > T];[322C > T] or p.[Arg108Cys];[Arg108Cys] located in *MMUT* (6 out of 42 patients, 14.3 %). Homozygous genotypes were found in 8 out of 31 MUTd patients (25.8 %), 5 out of 5 MMAAd patients (100 %, one of whom had a homozygous genotype for VUS), and 1 out of 2 MMABd patients (50 %) (Table 1).

Two patients (ID iMMA07 and iMMA08) bearing monoallelic genotypes for the p.(Arg108Cys) variant were found. Interestingly, one patient (ID iMMA09) carried a double heterozygous genotype consisting of the variants p.(Arg108Cys) in *MMUT* and c.733 + 1G > A in *MMAA*.

### 3.2 *MMUT* Hispanic variants

The variants previously identified in the Hispanic population by Worgan et al. [14], including c.322C > T or p.(Arg108Cys); c.280G > A or p.(Gly94Arg), c.970G > A or p.(Ala324Thr), and c.1022dupA or (Asn341Lysfs*20), were found in 22 patients in the present study, in either a homozygous, monoallelic heterozygous, compound heterozygous or double heterozygous state (Table 1). Considering the patient with the novel variants, in total we have 23 patients with Hispanic variants. The neonatal form of the disease was observed in 16 of these 23 patients (70 %). A more severe neonatal typical multisystemic presentation was noted in patients with the homozygous genotype constituted by the c.322C > T or p.(Arg108Cys) variant compared with those with only one c.322C > T or p.(Arg108Cys) allele.

### 3.3 Clinical and biochemical characteristics of Mexican patients with iMMA

Consanguinity and endogamy were documented in 7 of the 42 families (16.7 %) and in 2 of the 42 families (4.7 %), respectively. In 10 families, 13 previously deceased siblings with similar clinical symptoms were documented, most of whom died during the neonatal period. In one family, both consanguinity and endogamy were recorded.

### 3.4 Perinatal collected data

Prematurity was observed in 4 out of 32 (12.5 %) MUTd patients and in 1 out of 6 MMAAd patients (16.7 %). Notably, 10 out of 42 patients (23.8 %) required hospitalization immediately after birth, of whom nine were MUTd and one was MMAAd. The reasons for hospitalization included low Apgar scores, cardiorespiratory depression, and poor neurological responses. However, in none of these cases was metabolic disease suspected.

Neonatal symptom onset was predominant in MUTd patients (20 out of 32, 62.5 %) and in most MMAAd patients (5 out of 6, 83.4 %). In contrast, MMABd and MMADHCd patients presented infantile symptom onset between 2 and 24 months.

The median age of the patients at symptom onset was four days (Q1: 0.03 months [1 day], Q3: 4.1 months), with a minimum of 1 day and a maximum of 12 months. Unfortunately, the median diagnostic duration was 7.17 months (Q1: 1.8 months, Q3: 9 months), with a maximum of 52.7 months.

### 3.5 Biochemical characteristics

The biochemical characteristics of the patients with iMMA at the time of diagnosis are shown in Table 2. No statistically significant differences were observed in the biomarker concentrations among the different types of iMMA.

**Table 2:** Main diagnostic biochemical biomarkers of iMMA in Mexican patients. The data are presented as medians (Q1–Q3).

	MUTd *N* = 31	MMAAd *N* = 6	MMABd N = 3	*MMUT*/*MMAA* double heterozygous N = 1	MMADHCd N = 1
C3 (3.9–5.7 μmol/L)	19.03 (7.7–33.2)	7.13 (6.2–11.1)	23.3 (4.7–28.3)	24.6	7.5
Glycine (559–767 μmol/L)	423.7 (346.6–865.9)	599.4 (350.0–710.0)	676.3 (248.4–913.8)	618.12	637.3
Urinary organic acid profile	Increased excretion of urinary methylmalonic and methylcitric acids and propionylglycine				

After biochemical diagnosis, all the patients started treatment for iMMA following international guidelines [1,22,23]. Considering molecular diagnosis, the proposal for treatment adjustments that should be made in 11 out of 42 patients (26.1 %) depended on the specific affected gene (Table S1).

### 3.6 *In silico* structural analysis of the *MMUT* variant p.(Arg108Cys)

**Fig. 3:**
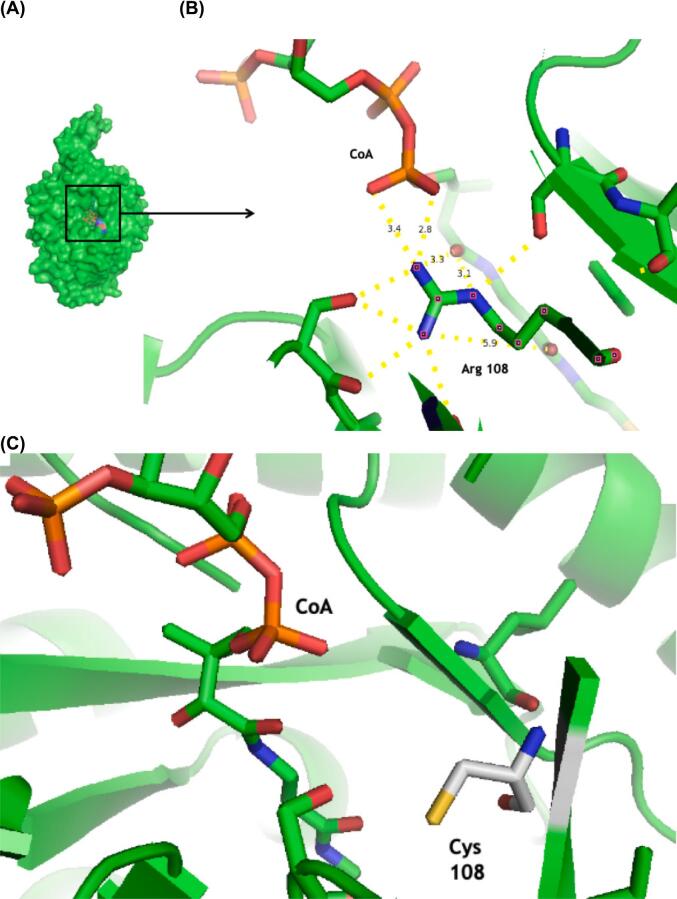
Crystallographic structure of human MUT. A). Monomer showing catalytic site cavity. B) Location of arginine 108 residue. C). The modeling of p.(Arg108Cys) pathogenic variant by *in silico* mutagenesis shows the possible loss of polar contacts with CoA moiety from the substrate.

To investigate the potential effect of the p.(Arg108Cys) variant on protein structure, *in silico* analysis was performed. In wild-type MUT, the arginine 108 residue is located at the substrate binding domain; in fact, it is in close contact with the substrate (mean distance 3.7 Å) (Fig. 3A), and the interaction between the amino groups of arginine residue 108 and the oxygen atoms from the phosphate and hydroxyl groups of the CoA molecule of the substrate is polar (Fig. 3B). When arginine, which has a longer lateral chain, is substituted by a cysteine residue, all these polar contacts with CoA are likely lost (Fig. 3C).

### 3.7 Detailed clinical and *postmortem* description of the patient bearing the genotype constituted by two novel *MMUT* variants

**Fig. 4:**
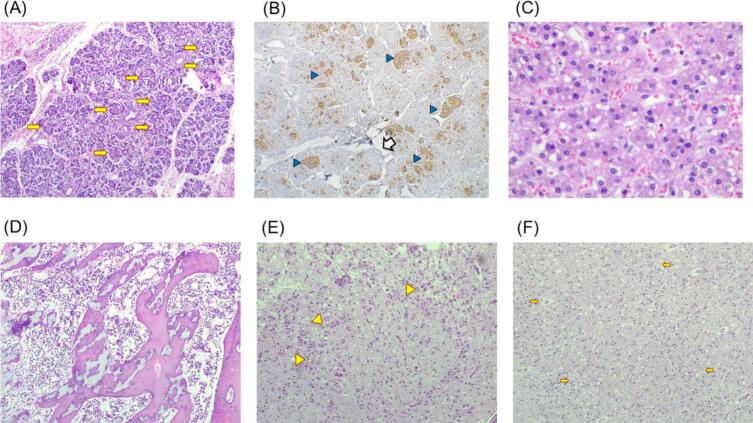
Histopathological autopsy findings in the patient (iMMA26) with the novel compound heterozygous genotype NM_000255.4(*MMUT*):c.[589G > A];[1476C > A] or p.[Ala197Thr];[Tyr492*]. (A) Photomicrograph (10×) of the pancreas showing the presence of pancreatic islets increased in number and size (arrows). (B) Photomicrograph (10×) of immunohistochemistry study (Chromogranin), which shows the pancreatic islets (arrowheads). (C) Photomicrograph (40×) showing liver tissue, with the presence of microvesicular steatosis (arrowheads). (D) Bone marrow (40×) showing accentuated hypoplasia of cellularity. (E) Stem midbrain (10×) showing the presence of Gliosis (Arrowheads). (F) Frontal brain region (10×) showing hypoxic cell retraction (arrows).

The novel genotype c.[589G > A];[1476C > A] or p.[Ala197Thr];[Tyr492*], was identified in a 4-day-old girl (ID iMMA26). She was the product of the fourth gestation of a healthy 31-year-old mother and a 34-year-old father. The couple had a history of one miscarriage and two deceased sons at the first days of life. The girl's symptoms initiated on the day she was born, and she presented with the typical clinical picture of MUTd. The patient exhibited a severe clinical course with metabolic acidosis, hyperammonemia, anemia, leukopenia, thrombocytopenia, jaundice and persistent hyperglycemia. She died at six days of age. Postmortem microscopic examination of the pancreas revealed hyperplasia and an increased number of islets of Langerhans, suggesting nesidioblastosis. Microvesicular steatosis was found in the liver. The bone marrow displayed marked hypocellularity, gliosis and hypoxic cell retraction were observed in the brain (Fig. 4).

### 3.8 Structural changes in MUT protein induced by the missense novel Mexican *MMUT* variant

The novel variant c.1476C > A or p.(Tyr492*) introduces a premature stop codon at the linker domain of the MUT protein. Owing to the nonsense nature of this variant, no structural model was created.

**Fig. 5:**
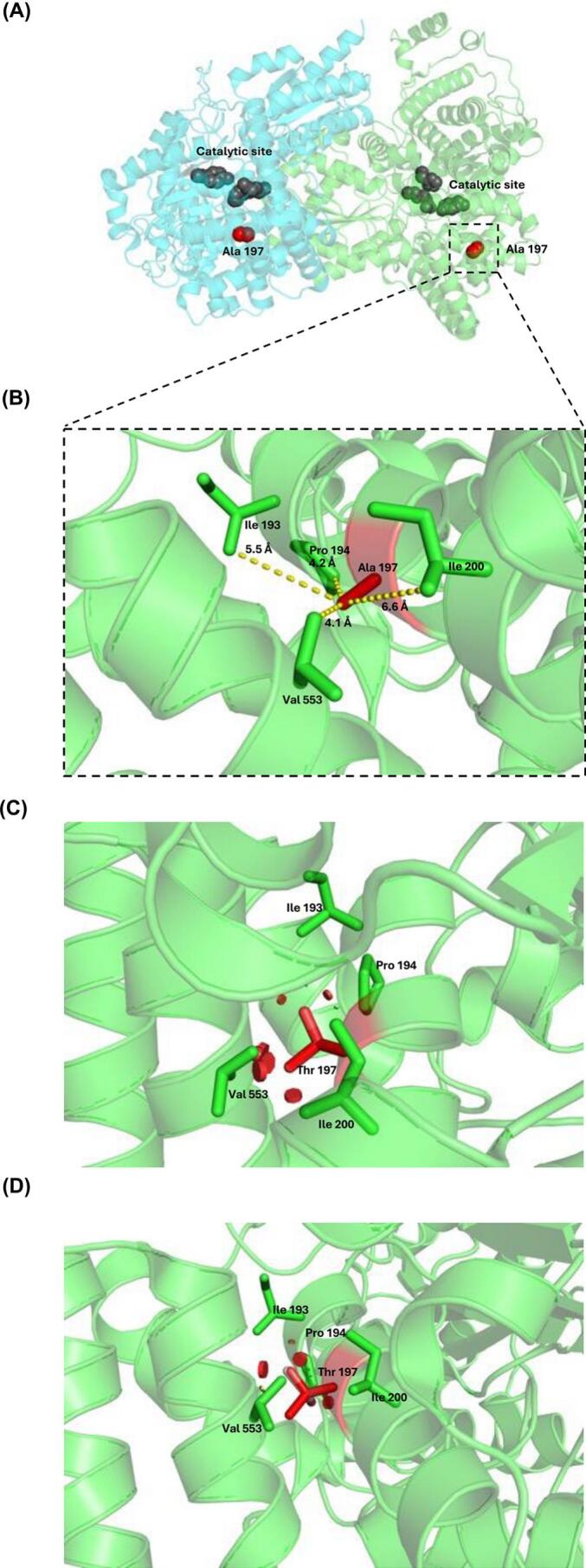
Localization of Ala 197 residue in the structure of methylmalonyl-CoA mutase. (A) General localization of Ala 197 residue. (B) Ala 197 residue is in close contact with Val 553, Pro 194, Ile 193, and Ile 200 residues within the same chain. (C, D) The replacement of Ala residue by Thr residue causes repulsive clashes which probably destabilize the structure of the protein.

The c.589G > A or p.(Ala197Thr) variant induces a nonconservative change involving the substitution of a hydrophobic amino acid by a polar uncharged amino acid in the catalytic domain of the enzyme, 21.3 Å from the catalytic pocket (Fig. 5A). This substitution is in proximity with the Val 553, Pro 194, Ile 193, and Ile 200 residues at 4.1 Å, 4.2 Å, 5.5 Å, and 6.6 Å, respectively (Fig. 5B). The abnormal substitution of the hydrophobic Ala residue for the polar Thr residue could lead to repulsive interactions with these neighboring residues, as illustrated in Figs. 5 C—D.

## 4 Discussion

Although iMMA is a well-known disease, reports on the clinical and genotypic landscape of patients with Latin American ancestry are scarce. Our study provides a detailed description of Mexican iMMA patients, highlighting that MUTd is the most common type (73.8 %). Consistent with findings from other authors studying larger cohorts of patients with MUTd [1,24,25], exons 2 and 3 were the most frequently affected exons, exhibiting the highest number of variants (Fig. 2C). Notably, we did not find any cases of *MCEE* in this cohort. MCEEd was first described as a mild form of iMMA [26], but to date, only a few cases have been described [27], including a study performed by Heuberger et al., where MCEEd was identified in 6.6 % of all iMMA cases (*N* = 10/105 patients) [28]. However, its worldwide prevalence is still unknown.

Our study revealed that most of our patients with iMMA presented with early-onset symptoms within the first days of life [29]. The clinical pictures of these patients were concordant with those reported in the literature [1]. We found that 17.5 % of these patients required hospitalization immediately after birth, but even an inborn error of metabolism was not suspected in any of those patients. Furthermore, this early clinical presentation poses a significant challenge for newborn screening programs, as symptoms may develop before the results are available [29,30]. For this reason, MMA has been classified as a time-critical condition in the US newborn screening panel (https://www.newsteps.org/media/8/download?inline).

In this cohort, the iMMA diagnosis was delayed, with patients experiencing a long diagnostic odyssey, with a median of 2.8 months (Q1 1 month, Q3 12.4 months). The clinical data of patients with MUTd are quite similar to those described in other populations, involving multiple systems, mainly the neurological, gastrointestinal, hematological, and immune systems [1].

Missense *MMUT* variants were the most prevalent in this study (11 out of 21, 52.4 %) (Fig. 2A), which agrees with findings from studies analyzing larger numbers of European (56.4 % [31]) and Chinese (60.9 % [32]) patients. Indels constitute the second most frequent type of pathogenic variant, representing 23.8 %, which is consistent with previously reported findings [31,32].

Concerning Hispanic variants, our data agrees with the findings of Worgan et al., who reported that c.322C > T or p.(Arg108Cys), c.280G > A or p.(Gly94Arg), and c.1022dupA or p.(Asn341Lysfs*20) are the most prevalent *MMUT* variants in Mexican patients [13]. With respect to the most frequently found Hispanic *MMUT* variant c.322C > T or p.(Arg108Cys), *in silico* modeling predicted that the substitution of an amino acid with different lateral chain lengths and polarities could decrease substrate interactions with amino acid residues from the active site during catalysis (Fig. 3), which could explain the severe phenotype observed in our homozygous patients. The interaction of the Arg108 residue with the substrate was previously described by Thomä and Leadlay in 1996 [33]. Functional studies are warranted to establish its definitive pathogenic, functional, and structural effects.

Regarding the two patients (Patient IDs iMMA07 and iMMA08, Table 1) biochemically and clinically classified with iMMA and bearing the monoallelic *MMUT* c.[322C > T];[=] genotype, their clinical exome results did not reveal the presence of CNVs in *MMUT*; thus, additional methodologies, such as whole-genome sequencing or RNA-Seq assays, could be employed to rule out deep intronic mutations or other eventual complex gene rearrangements [34].

**Table 1:** Genotypes in patients with iMMA (*n* = 42), presented by frequency and type of disease.

Genotype (cDNA)	Genotype (Protein)	Number of patients	Patients ID
***MMUT***			
c.[322C > T];[322C > T]	p.[Arg108Cys];[Arg108Cys]	6	iMMA01, iMMA02, iMMA03, iMMA04, iMMA05, iMMA06
c.[322C > T];[=]	p.[Arg108Cys];[=]	2	iMMA07, iMMA08
c.[322C > T];[280G > A]	p.[Arg108Cys];[Gly94Arg]	3	iMMA10, iMMA11, iMMA12
c.[322C > T];[1022dupA]	p.[Arg108Cys];[Asn341Lysfs*20]	2	iMMA13, iMMA14
c.[607G > A];[607G > A]	p.[Gly203Arg];[Gly203Arg]	2	iMMA15, iMMA16
c.[322C > T];[385 + 3insTAAGGGT]	p.[Arg108Cys];[?]	1	iMMA17
c.[322C > T];[91C > T]	p.[Arg108Cys];[Arg31*]	1	iMMA18
c.[322C > T];[1739_1746delATCGAATG]	p.[Arg108Cys];[Asp580fs]	1	iMMA19
c.[322C > T];[2080C > T]	p.[Arg108Cys];[Arg694Trp]	1	iMMA20
c.[322C > T];[1531C > T]	p.[Arg108Cys];[Arg511*]	1	iMMA21
c.[280G > A];[280G > A]	p.[Gly94Arg];[Gly94Arg]	1	iMMA22
c.[280G > A];[2179C > T]	p.[Gly94Arg];[Arg727*]	1	iMMA23
c.[330 T > G];[330 T > G]	p.[Tyr110*];[Tyr110*]	1	iMMA24
c.[406G > T];[1891delG]	p.[Val136Phe];[Ala631Glnfsx17]	1	iMMA25
**c.[589G** **>** **A];[1476C** **>** **A]**	p.[Ala197Thr];[Tyr492*]	1	iMMA26
c.[671-678dupAATTTATG];[682C > T]	p.[Val227Asnfsx16];[Arg228*]	1	iMMA27
c.[682C > T];[970G > A]	p.[Arg228*];[Ala324Thr]	1	iMMA28
c.[842 T > C];[842 T > C]	p.[Leu281Ser];[Leu281Ser]	1	iMMA29
c.[1445-1G > T];[1445-1G > T]	p.[?];[?]	1	iMMA30
c.[1846C > T];[1846C > T]	p.[Arg616Cys];[Arg616Cys]	1	iMMA31
c.[2080C > T];[2080C > T]	p.[Arg694Trp];[Arg694Trp]	1	iMMA32

***MMAA***			
c.[443 T > A];[443 T > A]	p.[Leu148*];[Leu148*]	2	iMMA33, iMMA34
c.[387C > A];[387C > A]	p.[Tyr129*];[Tyr129*]	1	iMMA35
c.[638C > T];[638C > T]	p.[Thr213Ile];[Thr213Ile]	1	iMMA36
c.[733 + 1G > A];[733 + 1G > A]	p.[?];[?]	1	iMMA37
c.[988C > T];[988C > T]	p.[Arg330*];[Arg330*]	1	iMMA38

***MMAB***			
c.[556C > T];[556C > T]	p.[Arg186Trp];[Arg186Trp]	2	iMMA39, iMMA40
c.[556C > T];[571C > G]	p.[Arg186Trp];[Arg191Gly]	1	iMMA41

***MMADHC***			
c.[472C > T];[160C > T]	p.[Arg158*];[Arg54*]	1	iMMA42

***MMUT/MMAA* double heterozygous**			
*MMUT* c.[322C > T];[=]	p.[Arg108Cys];[=]	1	iMMA09^**A**^
*MMAA* c.[733 + 1G > A];[=]	p.[?];[=]		

Variants previously found in Hispanic population are highlighted in red [13], new variants are highlighted in bold. ^**A**^Double heterozygosity. Patients with consanguineous or endogamic parents are highlighted in blue.

Interestingly, we identified a patient who was the product of the first pregnancy of a second-degree consanguineous couple (uncle and niece) carrying a double heterozygous genotype involving the *MMUT* variant p.(Arg108Cys) and the pathogenic splice site variant c.733 + 1G > A in the *MMAA* gene (ID iMMA09). This patient presented a clinical picture and a biochemical profile highly suggestive of MUTd, with a very high blood concentration of C3 (24.6 μmol/L, reference range 3.9–5.7 μmol/L), as well as increased excretion of urinary methylmalonic and methylcitric acids and propionylglycine (Table 2). Owing to the highly indicative clinical and biochemical picture of MUTd in this patient, the possibility of a synergistic heterozygosity phenomenon, which could arise by compromising the efficiency of two or more catalytic steps in complex metabolic pathways [35], should be considered. Similarly, Vockley et al. described a synergistic heterozygosity effect in patients with glycogen storage disorders (GSDs), consisting of two unrelated families with a clinical and biochemical diagnosis of GSDs involving simultaneous heterozygous variants in three different genes involved in the glycogen metabolic pathway, *PYGL* (GSD VI), *AGL* (GSD III), and *GBE1* (GSD IV) [35].

To the best of our knowledge, synergistic heterozygosity has not been previously identified as a genetic mechanism leading to iMMA; however, the catalytic mechanism, recently detailed in greater profundity by the findings of Mascarenhas et al., could support our hypothesis [8]. In this mechanism, during its catalytic activity, MUT utilizes AdoCbl as a cofactor for the generation of radicals that allow isomerization of the substrate methylmalonyl-CoA to succinyl-CoA. During catalysis, AdoCbl becomes oxidized and useless for new catalytic cycles; thus, the GTPase-MMAA participates as a chaperone, assisting in the recycling of the oxidized cofactor and the replenishment of a reduced cofactor to allow MUT to be available for further catalytic cycles [20,36]. This highly complex mechanism in which the participation of MMAA is indispensable to assist MUT, makes it suitable to propose the hypothesis of synergistic heterozygosity as a possible inheritance mechanism underlying iMMA, in which the interaction of an affected subunit of MUT with an affected subunit of MMAA during catalysis could compromise the overall metabolic pathway, leading to the MUTd phenotype in our double heterozygous patient. This hypothesis deserves further experimental demonstration, after discarding any other variant outside of the exome.

The *postmortem* analysis of the newborn patient with the novel *MMUT* genotype revealed liver steatosis and damage to the brain and midbrain, consistent with previous reports [1,7], as well as bone marrow hypocellularity [37], and hyperplasia with increased number of pancreatic islets of Langerhans (Figs. 4 A-B), suggestive of noninsulinoma pancreatogenous hypoglycemia syndrome (NIPHS), formerly known as nesidioblastosis. Genetic variants related to NIPHS were also explored (Supplementary Table S2 [38]), with no coincidental findings. The structural *in silico* prediction of the protein resulting from c.589G > A or p.(Ala197Thr) present in this patient suggests a potential derangement of the vicinity of the Ala 197 residue (Fig. 5). The consequences of this possible structural damage deserve further functional studies, such as molecular dynamics, and determination of the kinetic parameters of the respective recombinant protein. Taken together, these structural findings in combination with the nonsense variant could explain the severity, early onset, and fatal outcome of the disease in this patient.

Finally, for patient iMMAA-36, who presented a biallelic *MMAA* homozygous genotype consisting of the VUS variant.638C > T or p.(Thr213Ile) (Fig. 2D), further functional studies must be conducted to reclassify the genotype and correlate it with the typical clinical picture and biochemical profile of MMAAd documented in this patient.

## 5 Conclusions

This study provides the first comprehensive clinical and genotypic characterization of the largest cohort of Mexican patients with iMMA, highlighting that MUTd is the most prevalent form. Our findings are consistent with reports from other regions, confirming that *MMUT* variants are the most common worldwide genetic cause of iMMA, particularly among patients of Hispanic ancestry. Notably, the study identified two novel *MMUT* variants in a compound heterozygous patient: c.[589G > A];[1476C > A] or p.[Ala197Thr];[Tyr492*], presenting with a severe neonatal course. These findings support the wide genotypic diversity associated with this disorder. The identification of a double *MMUT* and *MMMA* heterozygous patient needs further assessment to confirm the possibility of synergistic heterozygosity as a complex and previously unreported inheritance pattern in this disease.

The results emphasize the challenges associated with early diagnosis, particularly in neonatal cases, where symptoms often present before newborn screening results are available. This underscores the need for improved diagnostic strategies to reduce diagnostic delay and the associated morbidity. The clinical outcomes observed, including severe multisystemic involvement and novel findings such as early pancreatic hyperplasia, further illustrate the complexity of iMMA and the need for tailored therapeutic approaches based on molecular diagnosis.

This study expands the current knowledge on the molecular spectrum of iMMA in the Mexican population and reinforces the importance of genetic analysis in guiding clinical management. Future studies should focus on exploring the functional impact of the two identified novel *MMUT* variants; investigating emerging phenotypic features, such as lipodystrophy, is also warranted.

## Funding

This research was funded by the Instituto Nacional de Pediatría, Secretaría de Salud (Recursos Fiscales 2022–2024, Programa E022 Investigación y Desarrollo Tecnológico en Salud, Ciudad de México, México, protocol numbers 052/2016 and 2024/032).

## Institutional review board statement

This study was conducted according to the guidelines of the Declaration of Helsinki and approved by the Institutional Review Board (Ethics, Research, and Biosafety Committees) of the National Institute of Pediatrics (Reference protocol numbers 052/2016 and 2024/032).

## CRediT authorship contribution statement

**Cynthia Fernández-Lainez:** Writing – review & editing, Writing – original draft, Visualization, Supervision, Software, Resources, Project administration, Methodology, Investigation, Funding acquisition, Formal analysis, Data curation, Conceptualization. **Marcela Vela-Amieva:** Writing – review & editing, Writing – original draft, Visualization, Validation, Software, Resources, Methodology, Investigation, Funding acquisition, Formal analysis, Data curation, Conceptualization. **Miriam Reyna-Fabián:** Writing – review & editing, Validation, Methodology, Formal analysis, Data curation. **Liliana Fernández-Hernández:** Writing – review & editing, Validation, Methodology, Formal analysis, Data curation. **Sara Guillén-López:** Writing – review & editing, Methodology, Investigation, Formal analysis. **Lizbeth López-Mejía:** Writing – review & editing, Methodology, Investigation, Formal analysis. **Miguel Ángel Alcántara-Ortigoza:** Writing – review & editing, Writing – original draft, Visualization, Validation, Resources, Investigation, Formal analysis, Data curation. **Ariadna González-del Angel:** Writing – review & editing, Writing – original draft, Visualization, Validation, Methodology, Formal analysis. **Rosa Itzel Carrillo-Nieto:** Writing – review & editing, Visualization, Methodology, Data curation. **Enrique Ortega-Valdez:** Writing – review & editing, Visualization, Methodology, Data curation. **Mauricio Rojas-Maruri:** Writing – review & editing, Validation, Methodology. **Cecilia Ridaura-Sanz:** Writing – review & editing, Visualization, Methodology, Formal analysis.

## Declaration of competing interest

None.

## Data Availability

Data will be made available on request.

## Appendix A Supplementary data

Supplementary material 1 Supplementary material 1

Supplementary material 2 Table S2. Genetic variants related to non-insulinoma pancreatogenous hypoglycemia syndrome. Supplementary material 2
