# Supplementary material for: Isolated methylmalonic acidemia in Mexico: Genotypic spectrum, report of two novel MMUT variants and a possible synergistic heterozygosity effect
Source: Mol Genet Metab Rep. 2024 Oct 16;41:101155. doi: 10.1016/j.ymgmr.2024.101155 (PMC11530693; doi:10.1016/j.ymgmr.2024.101155)
Supplement: Supplementary material 1 [file mmc1.docx]

Table S1. Proposal of adjustment to the treatment of iMMA patients after clinical exome studies.

|  | **No change needed** | **Treatment adjustment proposed** | | | | |
| --- | --- | --- | --- | --- | --- | --- |
|  | **MMUT**  **(N = 31)** | **MMAA**  **(N = 6)** | **MMUT/MMAA**  **(N = 1)** | **MMAB ^B^**  **(N = 3)** | **MMADHC**  **(N = 1)** | **MCEE** |
| **Patients ID** | iMMA01-iMMA08, iMMA10-iMMA32 | iMMA33-iMMA38 | iMMA09 | iMMA39-iMMA41 | iMMA42 | No patients found. |
| **Hydroxy-cobalamin (OHCbl)** | Mut^0^: Not responsive to injectable B_12_ therapy. Mut ^–^ : rarely B_12_-responsive.  In responder patients 1 mg IM daily [22]. | 1 mg IM daily [22, 23]. | | | | Not responsive to injectable B_12_ therapy [1]. |
| **Levocarnitine [1, 22]** | 50-100 mg/kg/d | | | | | |
| **Dietary treatment** | Dietary Reference Intakes for protein requirements.  Low natural protein diet.  Use medical foods (methionine- and valine-free, low in isoleucine, and threonine) in moderation only when necessary to complete protein requirements [1, 22]. | Dietary Reference Intakes for protein requirements [22]. | Dietary Reference Intakes for protein requirements.  Low natural protein diet.  Use medical foods (methionine- and valine-free, low in isoleucine, and threonine) in moderation only when necessary to complete protein requirements [1, 22]. | Dietary Reference Intakes for protein requirements [22]. | | |

**^B^** In responder patients. Response to hydroxy-cobalamin is defined as a mean decrease of urine/plasma methylmalonic acid concentration of > 50% [22].
